# Identification of ferroptosis-related genes and predicted overall survival in patients with burns

**DOI:** 10.3389/fsurg.2022.1060036

**Published:** 2023-01-09

**Authors:** Mingjian Zhao, Yetong Zhang, Hongliang Zhao

**Affiliations:** ^1^ Graduate School, Dalian Medical University, Dalian, China; ^2^ Department of Burns and Plastic Surgery, Miyun Hospital, Capital Medical University, Beijing, China

**Keywords:** ferroptosis-related genes, predicted, burn, patient, survival

## Abstract

**Introduction:**

Burns are a common trauma associated with considerable mortality and morbidity. Although a lot is known regarding burns' pathogenesis, the involvement of ferroptosis is uncertain. Here, we aimed to explore vital ferroptosis-related genes and molecules in burns, through bioinformatics analysis, to uncover new effective therapeutic targets.

**Methods:**

The FerrDb database was used to acquire ferroptosis-related genes and GSE19743 was downloaded from Gene Expression Omnibus (GEO), a dataset with analysis of control and burned individuals. Hub genes were selected with Cytoscape software, and Gene Ontology (GO), and Kyoto Encyclopedia of Genes and Genomes (KEGG) enrichment analyses were conducted. Cox proportional hazard function and Kaplan-Meier survival analyses were implemented to screen prognosis-related genes. Additionally, the miRWalk database was used to acquire the miRNAs relevant to our hub genes function and analyzed for enrichment.

**Result:**

We identified 64 differentially expressed genes and through the intersection with ferroptosis-related genes, 10 were selected as hub genes. GO analysis revealed that the hub genes' most enriched activities were response to oxidative stress, pyridine-containing compound metabolic processes, and reactive oxygen species metabolic processes. KEGG pathways' analysis showed that these overlapped genes were enriched in several pathways, namely, in VEGF signaling. Furthermore, the molecular miRNA functions significantly enriched were signal transduction and cell communication, namely, the biological pathways of the glypican pathway and the ErbB receptor signaling network. SLC40A1 and GPT2 genes were found to be associated with overall survival, suggesting an important role in burn prognosis.

**Discussion:**

This study may improve our understanding of the underlying burn mechanisms and provide a new direction for the prevention of poor outcomes, advances in burns treatment, and drug development.

## Introduction

Burns are severe public health incidents causing approximately 265,000 deaths worldwide. Although the incidence of burns in developed countries decreases yearly, high morbidity and mortality are still observed in developing countries (1). Symptoms such as a hypermetabolic state, acute kidney injury, and septicemia are life-threatening (2) in patients with severe burns. Crucially, these types of injuries also affect the patient's mental health and quality of life (3). Contractures, hypertrophic scarring, thermoregulation dysfunction, and other complications require long-term expensive rehabilitation, which delays or even prevents the return to a normal life (4). Therefore, finding effective therapeutic targets for burns has life-changing potential.

Ferroptosis was first proposed by Dixon et al. in 2012 as a metabolic dysfunction caused by iron-dependent lipid peroxidation and consequent accumulation of reactive oxygen species (ROS), leading to cell death (5, 6). This process is regulated by several cellular metabolic events, such as redox homeostasis, mitochondrial activity, iron handling, and metabolism of amino acids, lipids, and sugars, as well as signaling pathways associated with the disease (7). In recent years, ferroptosis has been correlated with the pathogenesis of several diseases, including cancer, inflammation, cardiovascular, kidney, and neurological diseases (8–15). To the best of our knowledge, there are only a few studies regarding ferroptosis in burns. However, it has been reported that reducing lipid peroxidation in burned tissues, thus inhibiting the formation of free radicals, can improve cardiac output, protect blood vessels (16), and promote wound angiogenesis and healing (16, 17). Therefore, exploring the occurrence of ferroptosis in burns may provide new therapeutic concepts.

In the present study, we used data analysis techniques to screen differentially expressed genes (DEGs) in burn patients’ blood samples and intersected this information with the FerrDb dataset to obtain ferroptosis differentially expressed genes (FDEGs) in burns. In addition, we selected 10 hub genes and explored their functional enrichments and prognosis potential to establish connections with treatment and outcomes. Our results will provide a better understanding of ferroptosis in burns and deliver new approaches for their clinical treatment.

## Materials and methods

### Gene expression microarray data

Ferroptosis-related genes (FRGs) were acquired from the FerrDb database and the expression profiles of burned patients from GEO (http://www.ncbi.nlm.nih.gov/geo/), encompassing microarrays, chips, and high-throughput gene expression data (18). The GSE19743 dataset, based on GPL570 (a platform for Affymetrix Human Genome U133 plus 2.0 Array), included 177 blood samples (63 healthy volunteers and 114 burn patients).

### Differential expression analysis of FRGs in burns

All data were processed in R software (version 3.6.0). The R package “GEOquery” was used to acquire GSE19743 from the GEO database (19). All probe sets were transformed by the platform accordingly into a gene symbol with annotated information. If it corresponds to the same gene, the maximum value of multiple probe sets was calculated. Data standardization and analysis of the two groups were carried out by the “limma” package, and the FRGs of |logFC| > 2/3 and *P* < 0.05 were retrieved from the obtained results and visualized with the “ggplot2” package.

### Identification of hub genes and protein–protein interaction establishment

STRING (www.string-db.org) was used to construct protein–protein interaction (PPI) networks and Cytoscape to adjust the graphics structure. A combined score of >0.4 was set as the cutoff criterion to avoid inaccurate PPIs (downloaded from STRING). Each node represents a gene and the edges represent correlations between them. The cytoHubba plugin for Cytoscape was used to explore the importance of each node in the biological networks; degree, closeness, and betweenness were analyzed and the top 10 nodes were selected. The common nodes were determined as hub genes. miRWalk 2.0 (www.uni-heidelberg.de) was also used to predict targeted pivotal miRNAs. The gene–miRNA interaction networks related to the hub genes were obtained from the miRWalk 2.0 software. In addition, hub gene–miRNA was collected from the miRWalk database. Furthermore, miRWalk and miRTarBase databases were used to obtain correlative miRNAs to ensure the accuracy and reliability of the results. *P* < 0.05 and 3′UTR as the target gene-binding regions were used in filtering the results.

### Enrichment analysis of FDEGs

The “cluster profiler” R package was used for enrichment analyses and FRG functions in burns were predicted based on the Gene Ontology (GO) function and the Kyoto Encyclopedia of Genes and Genomes (KEGG) pathway (20). The GO database (http://www.geneontology.org) contains three categories: molecular function (MF), biological processes (BP), and cellular components (CC). The KEGG database (http://www.genome.ad.jp/kegg/) includes chemical, genomic, and systemic function information. The *P* < 0.05 was defined as the criterion to determine the meaningfully enriched GO terms and KEGG pathways. The biological pathways of the miRNAs selected were analyzed in an enrichment analysis tool, Funrich 3.13.

### Construction of the prognostic signature of FDEGs in burns

Kaplan–Meier survival analysis and Cox proportional hazards regression analysis were performed with R packages “survival” and “survminer,” respectively. The “survival” package was used to analyze survival data and the “survminer” was used for result visualization. A forest plot was constructed with the R package “ggplot2.” In addition, the acquired prognosis-related genes were enriched using Gene Set Enrichment Analysis (GSEA) and Gene Set Variation Analysis (GSVA), employing the “cluster profiler” R package and the GSVA package, respectively (21).

### Immune infiltration landscape analysis

The immune infiltration landscape of blood samples from burn patients and healthy volunteers was investigated with “CIBERSORT R script v1.03” through the evaluation of 22 types of immune cells.

### Statistical analysis

First, burn patients in GSE19743 were constructed using a descriptive statistical analysis. Frequency and proportion were employed to describe categorical variables and mean ± standard deviation was used to define continuous variables. FRG expressions in burn patients were analyzed with a Wilcoxon rank sum test to determine statistical differences. The Benjamini–Hochberg method was used for multiple corrections and FDR < 0.05 was the standard for statistical significance. All statistical analyses were performed in R software, *P* values were two-sided and *P* < 0.05 was considered statistically significant.

## Results

### Differential expression analysis of FDEGs in burn

**Figure 1:**
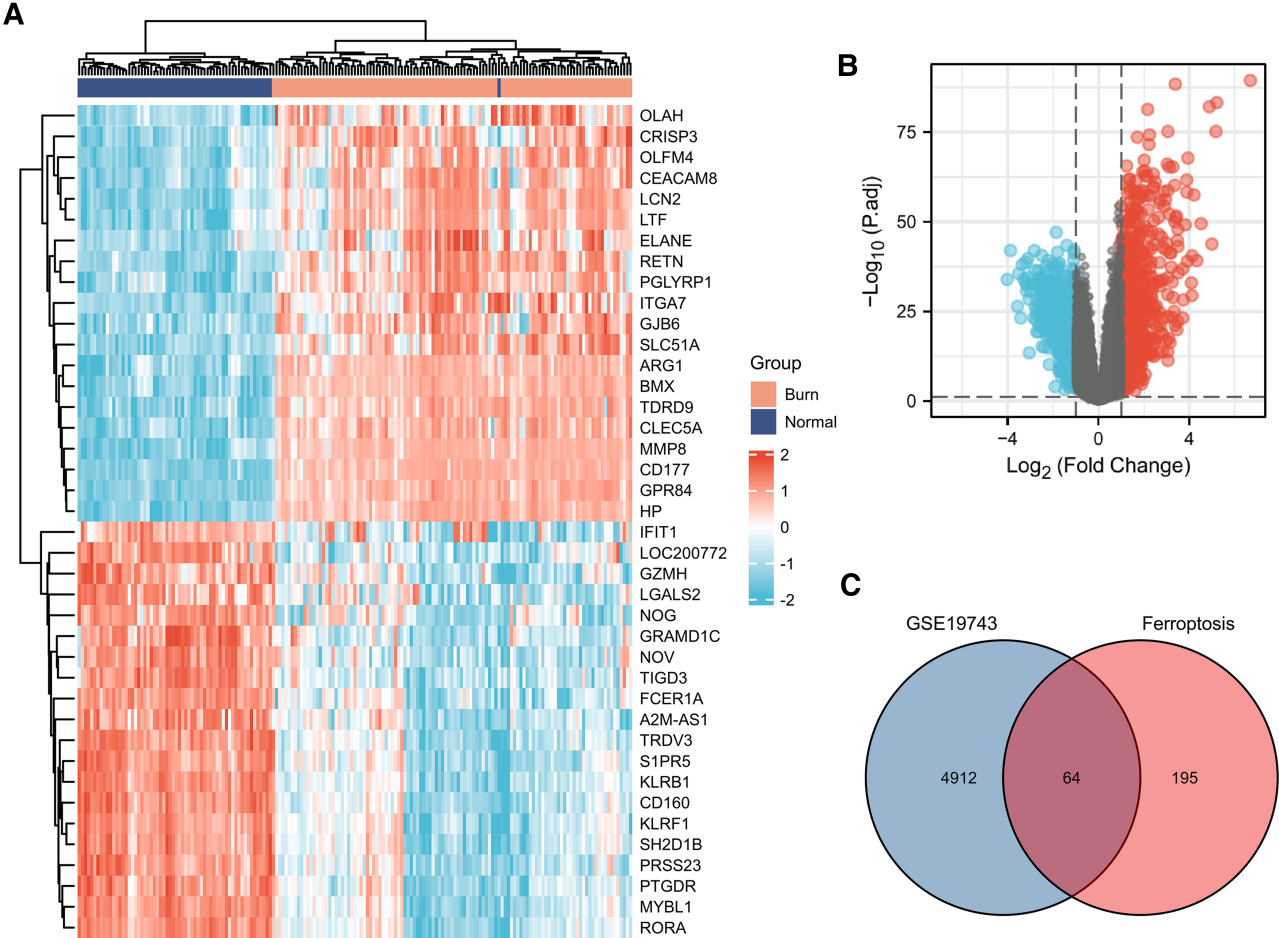
(**A**) Heatmap of the 40 differentially expressed genes in the GSE19743 database. (**B**) Volcano plot of differentially expressed genes in the GSE19743 database. (**C**) Venn plots of GSE19743 differentially expressed genes and ferroptosis-related genes.

A total of 259 FRGs were obtained from FerrDb. Through GSE19743 dataset analysis, employing logFC > 2/3 and *P* < 0.05 as screening criterion, we obtained 4,976 differentially expressed genes between burned and healthy groups. Crossing these two datasets, we obtained a total of 64 FDEGs (Figure 1, Table 1). The GSE37069 dataset was selected for validation. The GSE37069 is also a well-established dataset, widely used for validation or training sets and the validation results are satisfactory. We will add this part in Figure 8B.

**Table 1:** Ferroptosis differentially expressed genes.

	Gene symbol
Ferroptosis driver gene	ALOX15, WIPI1, PGD, EPAS1, DPP4, IFNG, MAPK14, ACVR1B, ACSF2, LPIN1, ATG13, LPCAT3, SLC38A1, NOX1, ALOX12, G6PD, TP53, IDH1, EMC2, GLS2, ULK1, LONP1, MAP1LC3A, CDO1, ACSL4, PEBP1, DUOX2, ULK2, MAPK3, SLC7A11, TNFAIP3
Ferroptosis suppressor gene	RRM2, AKR1C3, SCD, CBS, MUC1, HSPB1, NF2, ACSL3, HELLS, SLC40A1
Ferroptosis unclassified gene	CAPG, MAFG, HIC1, PLIN4, SLC2A3, PSAT1, STEAP3, SLC2A8, DRD4, AURKA, HAMP, SLC2A6 SLC7A5, HNF4A, ARRDC3, TAZ, SLC1A4, TUBE1 VLDLR, KLHL24, GPT2, EIF2AK4
Ferroptosis marker gene	PTGS2

### Protein–protein interaction network establishment and identification of hub genes

**Figure 2:**
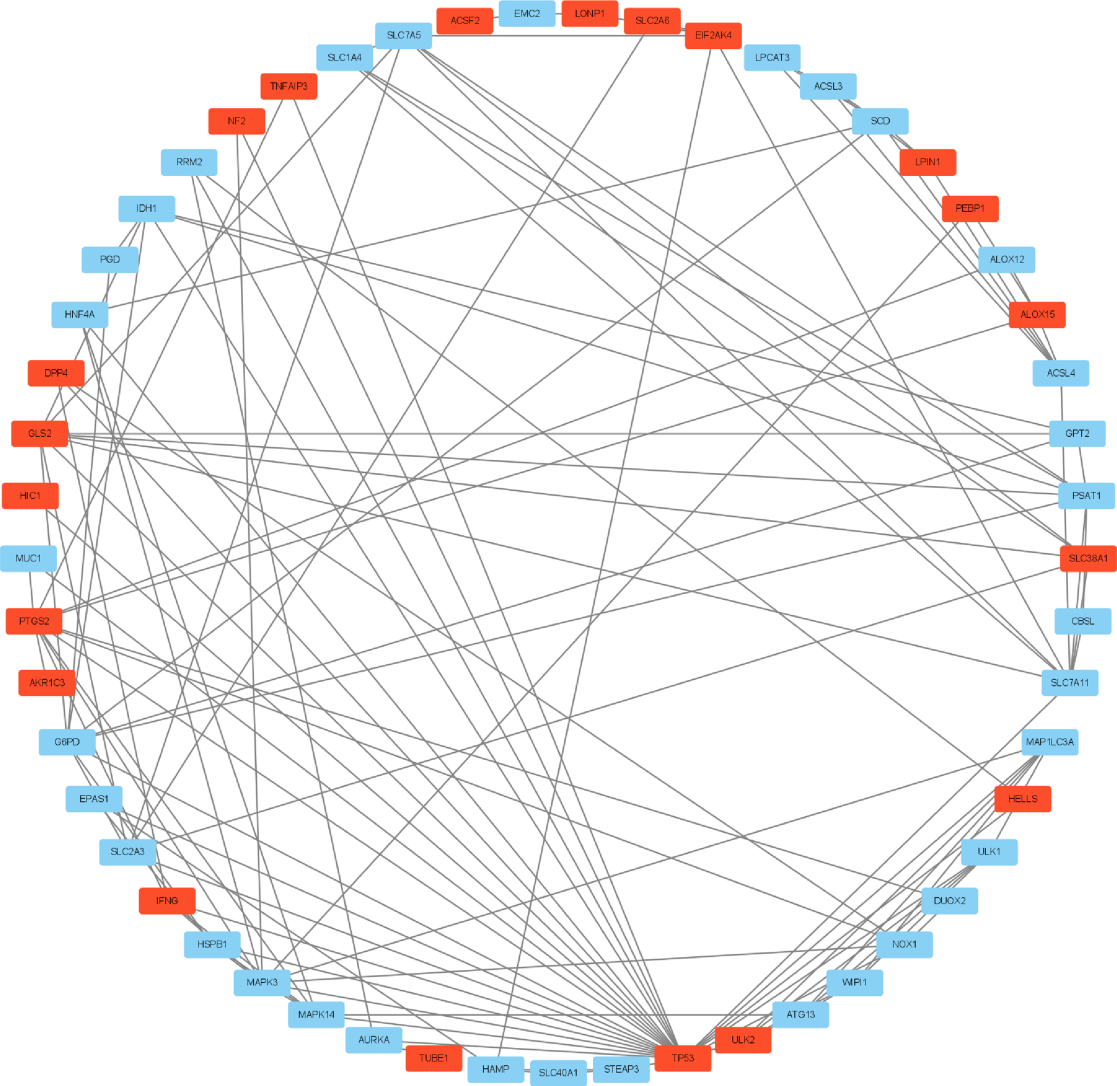
Ferroptosis-related 64 differentially expressed genes. Downregulated genes are plotted in red and upregulated genes in blue.

**Figure 3:**
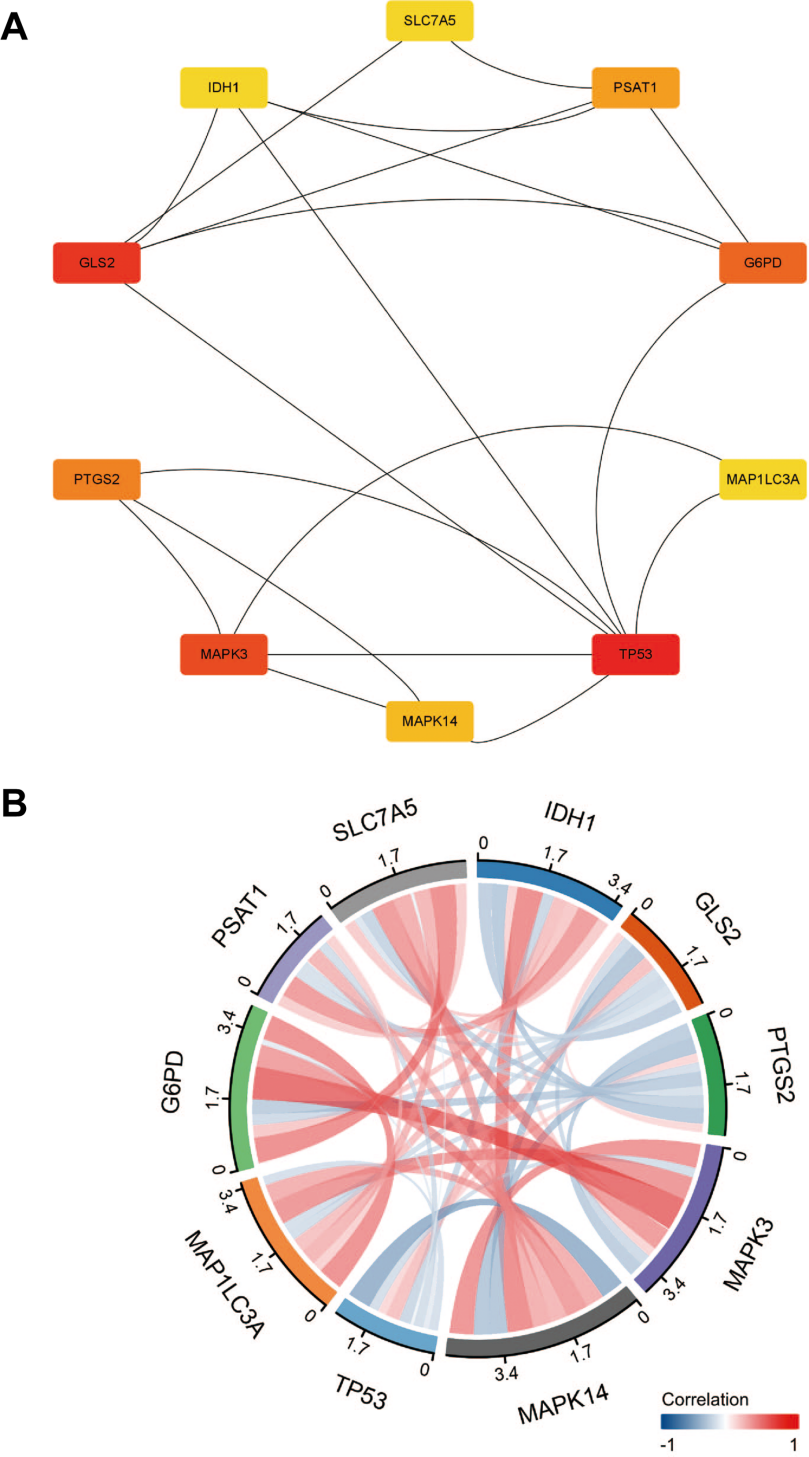
(**A**) Identified 10 hub genes. (**B**) Hub genes correlation chords plot.

**Figure 4:**
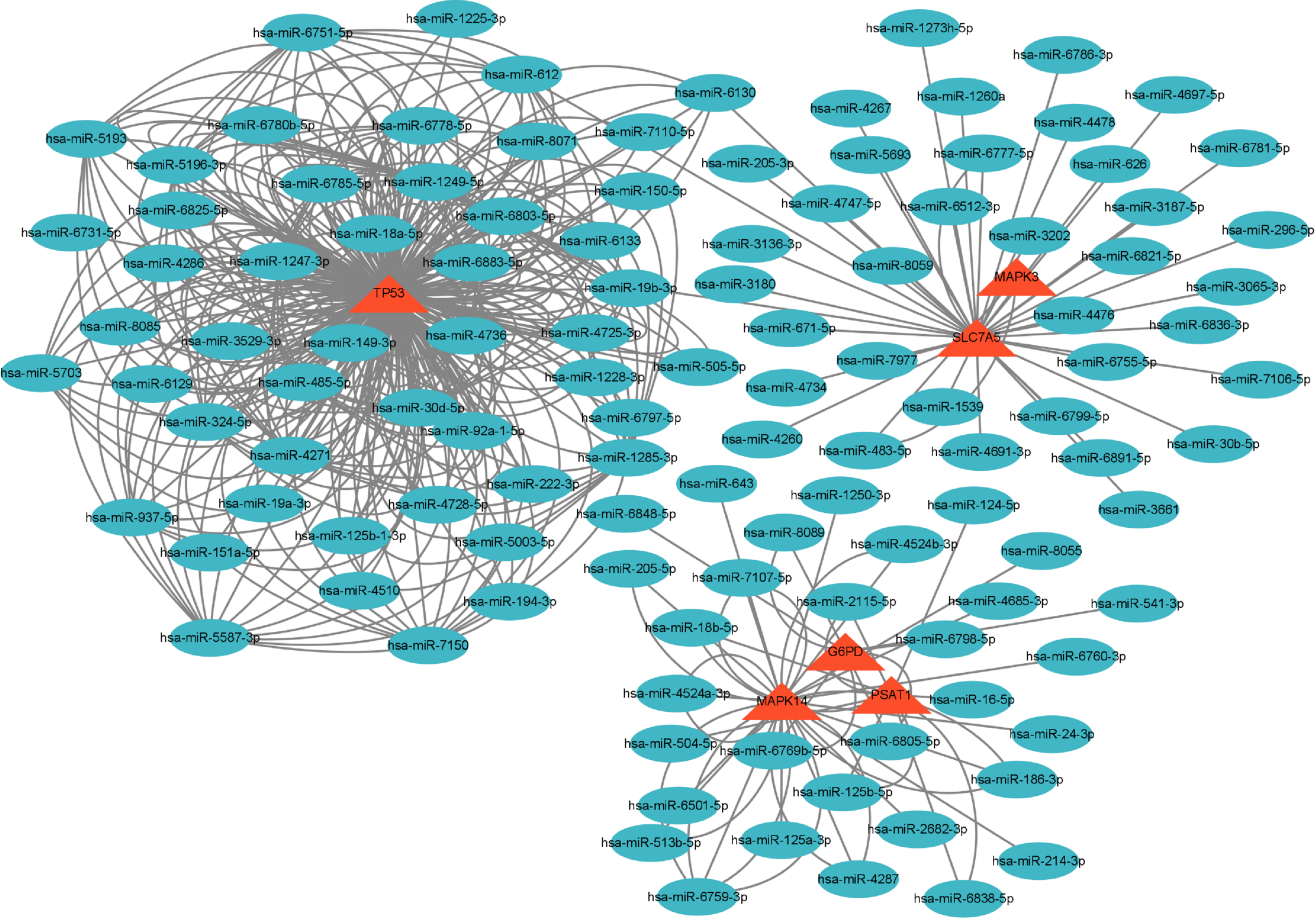
Gene–miRNA network. The red triangle represents the gene, and the green oval shape represents the miRNA.

A graph containing 53 nodes (genes) and 122 edges (gene interactions) was obtained and further analyzed using cytoHubba for key gene selection based on the number of nodes and edges. A total of 10 genes were selected as hub genes. In addition, the hub gene-related miRNA network, retrieved by the miRWalk database, provided a total of 122 nodes and 410 edges (Figures 2–4) and Table 4. The obtained hub genes were validated in the GSE37069 dataset.

### Enrichment analysis of FDEGs

**Figure 5:**
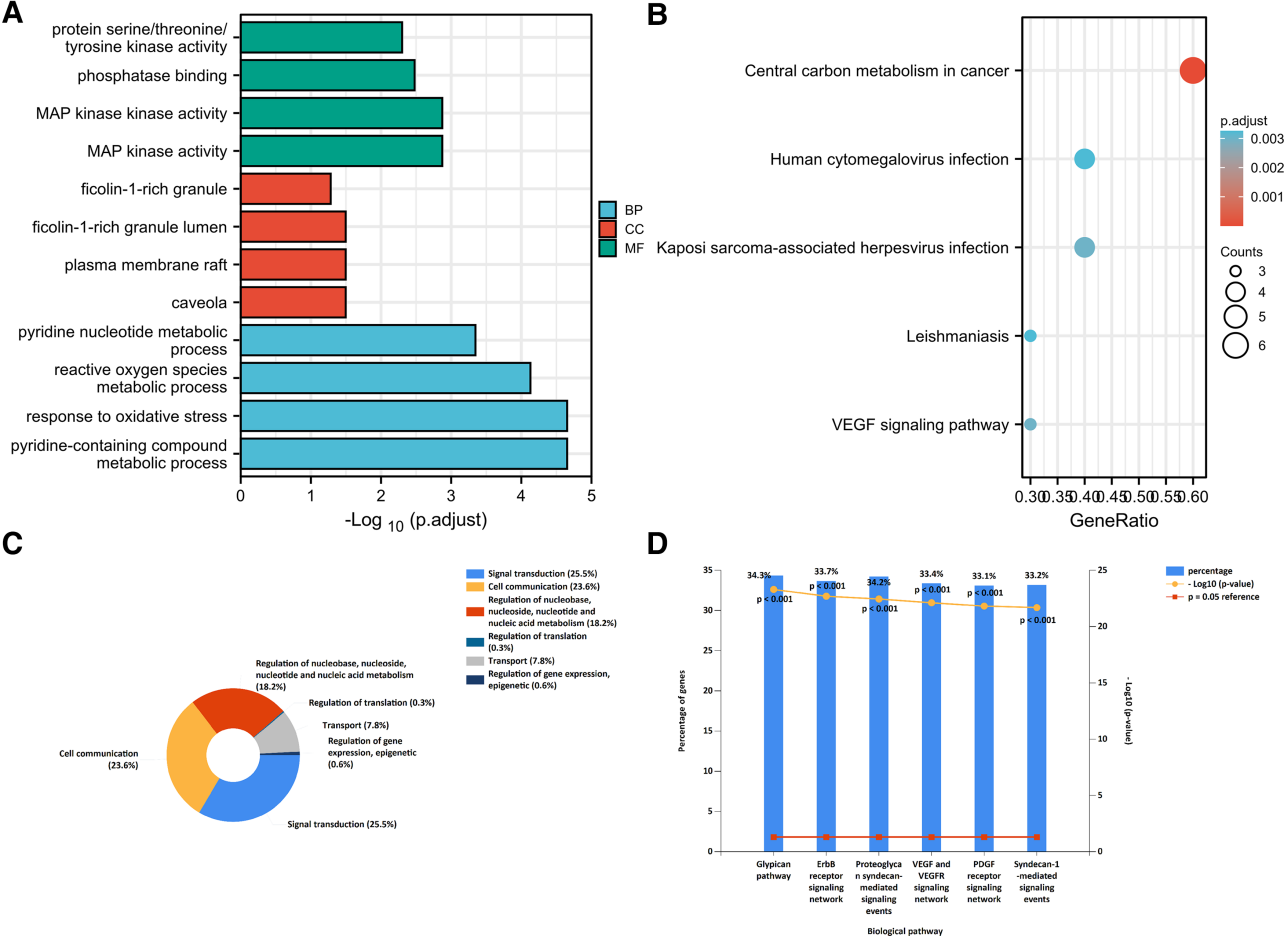
Enrichment analysis: (**A**) GO analysis; (**B**) KEGG analysis; (**C**) miRNA function enrichment; (**D**) miRNA biological pathway enrichment.

The “cluster profiler” package was used to analyze the GO function and KEGG enrichment to explore the mechanisms and functions of the FDEGs in burns. The results showed that in GO-BP, FDEGs were mainly related to oxidative stress, pyridine-containing compound metabolic processes, and reactive oxygen species metabolic processes. GO-CC analysis showed FDEGs association with caveola, plasma membrane raft, ficolin-1-rich granule lumen, ficolin-1-rich granule, and late endosome, among others. GO-MF analysis showed FDEGs association with mitogen-activated protein (MAP) kinase activity, phosphatase binding, protein serine/threonine/tyrosine kinase activity, NADP binding, etc. The top five enriched KEGG pathways for FDEGs were central carbon metabolism in cancer, Kaposi sarcoma-associated herpesvirus infection, VEGF signaling, human cytomegalovirus infection, and leishmaniasis (Figures 5A,B, Table 2).

**Table 2:** Go and KEGG enrichment analysis of Hub genes.

Ontology	ID	Description	GeneRatio	BgRatio	*P* value	*P* adjusted	*q* value
BP	GO:0072524	Pyridine-containing compound metabolic process	5/10	195/18,670	2.85 × 10^−8^	2.22 × 10^−5^	8.57 × 10^−6^
BP	GO:0006979	Response to oxidative stress	6/10	451/18,670	3.72 × 10^−8^	2.22 × 10^−5^	8.57 × 10^−6^
BP	GO:0072593	Reactive oxygen species metabolic process	5/10	284/18,670	1.86 × 10^−7^	7.42 × 10^−5^	2.86 × 10^−5^
BP	GO:0019362	Pyridine nucleotide metabolic process	4/10	189/18,670	2.04 × 10^−6^	4.50 × 10^−4^	1.74 × 10^−4^
CC	GO:0005901	Caveola	2/10	80/19,717	7.16 × 10^−4^	0.032	0.017
CC	GO:0044853	Plasma membrane raft	2/10	109/19,717	0.001	0.032	0.017
CC	GO:1904813	Ficolin-1-rich granule lumen	2/10	124/19,717	0.002	0.032	0.017
CC	GO:0101002	Ficolin-1-rich granule	2/10	185/19,717	0.004	0.052	0.028
MF	GO:0004707	MAP kinase activity	2/10	14/17,697	2.61 × 10^−5^	0.001	5.60 × 10^−4^
MF	GO:0004708	MAP kinase kinase activity	2/10	16/17,697	3.43 × 10^−5^	0.001	5.60 × 10^−4^
MF	GO:0019902	Phosphatase binding	3/10	185/17,697	1.28 × 10^−4^	0.003	0.001
MF	GO:0004712	Protein serine/threonine/tyrosine kinase activity	2/10	43/17,697	2.56 × 10^−4^	0.005	0.002

BP, biological processes; CC, cellular components; MF, molecular function.

A total of 410 miRNA were uploaded to the enrichment analysis tool Funrich; miRNA-associated molecular functions significantly enriched were cell communication, signal transduction, nucleoside, regulation of nucleobase, nucleotide, and nucleic acid metabolism. Proteoglycan syndecan-mediated signaling events, the ErbB receptor signaling network, and the glypican pathway were the biological pathways found to be enriched in this dataset (Figures 5C,D).

### Construction of the prognostic signature of FDEGs in burn

**Figure 6:**
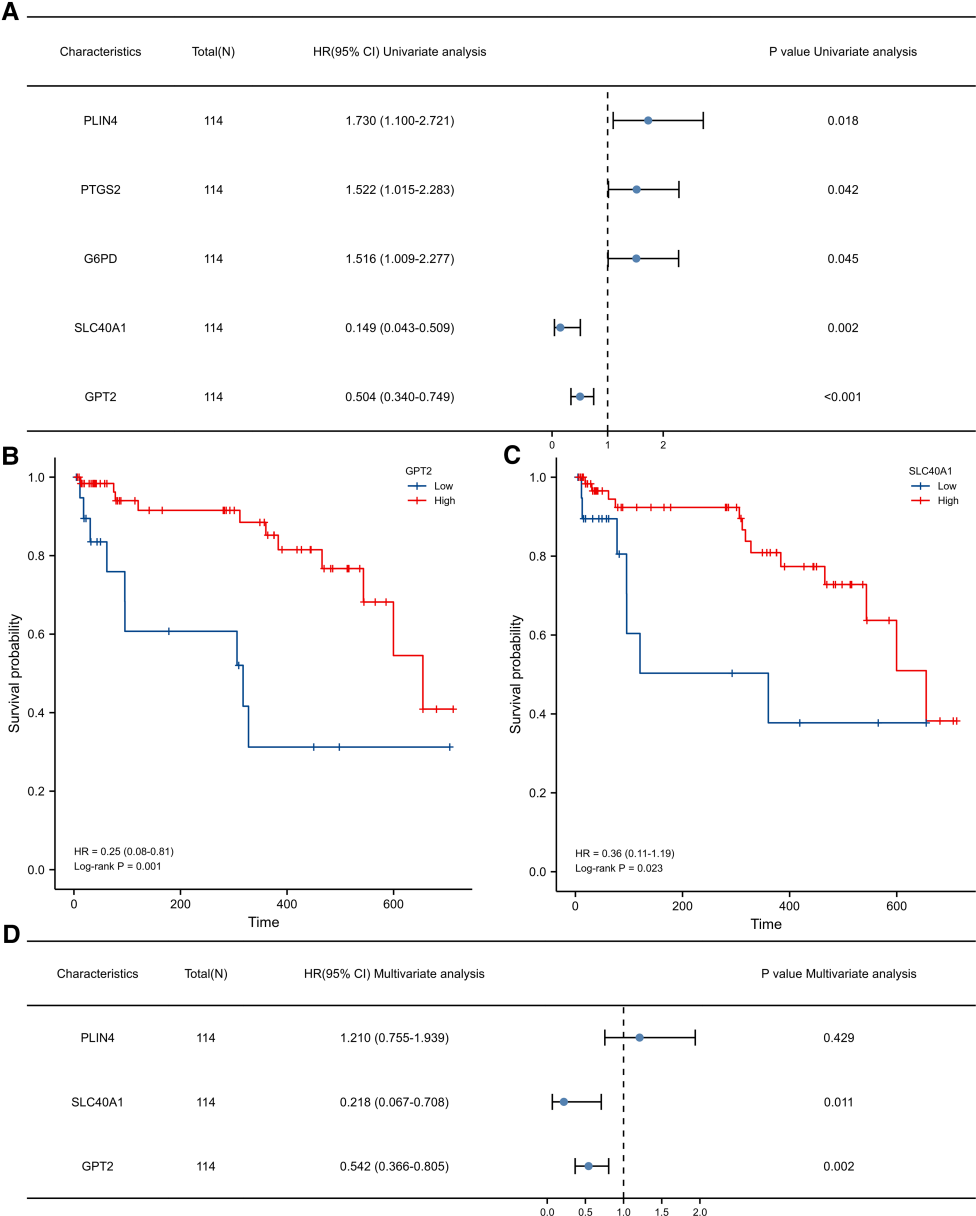
(**A**) Univariate Cox proportional hazard regression model. K–M survival analysis for the relationship between gene expression levels. (**B**) GPT2 and (**C**) SLC40A1 and prognosis. (**D**) Multivariate Cox proportional hazard regression model.

**Figure 7:**
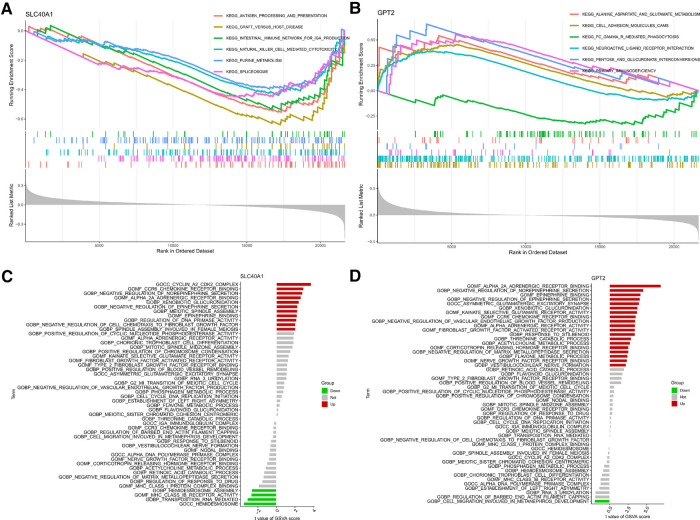
GPT2 and SLC40A1-related enrichment analysis: (**A,B**) GSEA and (**C,D**) GSVA analysis. GSEA, Gene Set Enrichment Analysis; GSVA, Gene Set Variation Analysis.

We further explored the relationship between FDEG expression and burn prognosis. We found that prostaglandin-endoperoxide synthase 2 (PTGS2) [HR = 1.522 (1.015–2.283), *P* = 0.042], G6PD [HR = 1.516 (1.009–2.277), *P* = 0.045], SLC40A1 [HR = 0.149 (0.043–0.509), *P* = 0.002], GPT2 [HR = 0.504 (0.340–0.749), *P* < 0.001], and PLIN4 [HR = 1.730 (1.100–2.721), *P* = 0.018] were highly correlated with overall survival through the univariate Cox proportional hazard regression model. With Kaplan–Meier survival analysis, SLC40A1 [HR = 0.36(0.11–1.19), *P* = 0.023], GPT2 [HR = 0.25(0.08–0.81), *P* = 0.001], and PLIN4 [HR = 3.52(1.46–8.48), *P* = 0.009] expression was further correlated with overall survival. Based on these results, we constructed a multivariate Cox proportional hazard regression model, and SLC40A1 [HR = 0.218 (0.067–0.708), *P* = 0.011] and GPT2 [HR = 0.542 (0.366–0.805), *P* = 0.002] were determined as biomarkers correlated to prognostic (Figure 6, Tables 3, 4). Furthermore, we added the enrichment analysis of GSEA and GSVA to explain the relationship between prognostic-related genes and burns (Figure 7).

**Table 3:** Univariate Cox proportional hazard regression model.

Characteristics	Total (*N*)	Univariate analysis	
		Hazard ratio (95% CI)	*P* value
PLIN4	114	1.730 (1.100–2.721)	**0.018**
PTGS2	114	1.522 (1.015–2.283)	**0**.**042**
G6PD	114	1.516 (1.009–2.277)	**0**.**045**
SLC40A1	114	0.149 (0.043–0.509)	**0**.**002**
GPT2	114	0.504 (0.340–0.749)	**<0**.**001**

Bold values indicate *P* < 0.05.

**Table 4:** Multivariate Cox proportional hazard regression model.

Characteristics	Total (*N*)	Multivariate analysis	
		Hazard ratio (95% CI)	*P* value
PLIN4	114	1.210 (0.755–1.939)	0.429
SLC40A1	114	0.218 (0.067–0.708)	**0.011**
GPT2	114	0.542 (0.366–0.805)	**0**.**002**

Bold values indicate *P* < 0.05.

**Table 5:** The relationship between miRNA and hub gene.

G6PD	hsa-miR-6798-5p, hsa-miR-8089, hsa-miR-541-3p, hsa-miR-6805-5p, hsa-miR-6848-5p, hsa-miR-6769b-5p, hsa-miR-6805-5p, hsa-miR-6769b-5p, hsa-miR-8089
TP53	hsa-miR-937-5p, hsa-miR-937-5p, hsa-miR-937-5p, hsa-miR-937-5p, hsa-miR-6133, hsa-miR-6133, hsa-miR-6133, hsa-miR-6133, hsa-miR-6133, hsa-miR-8071, hsa-miR-8071, hsa-miR-8071, hsa-miR-612, hsa-miR-6797-5p, hsa-miR-6883-5p, hsa-miR-7150, hsa-miR-18a-5p, hsa-miR-19b-3p, hsa-miR-125b-1-3p, hsa-miR-149-3p, etc.
MAPK3	hsa-miR-483-5p, hsa-miR-483-5p
MAPK14	hsa-miR-24-3p, hsa-miR-205-5p, hsa-miR-125a-3p, hsa-miR-4524a-3p, hsa-miR-4524b-3p, hsa-miR-6759-3p, hsa-miR-6760-3p, hsa-miR-7107-5p, hsa-miR-186-3p, hsa-miR-4287, hsa-miR-4524a-3p, hsa-miR-6759-3p, hsa-miR-205-5p, hsa-miR-214-3p, hsa-miR-186-3p, hsa-miR-643, hsa-miR-513b-5p, hsa-miR-4287, hsa-miR-4524a-3p, hsa-miR-4524b-3p, etc.
SLC7A5	hsa-miR-4691-3p, hsa-miR-6777-5p, hsa-miR-205-3p, hsa-miR-30b-5p, hsa-miR-296-5p,hsa-miR-612, hsa-miR-626, hsa-miR-671-5p, hsa-miR-1260a, hsa-miR-1539, hsa-miR-3136-3p, hsa-miR-3065-3p, hsa-miR-3187-5p, hsa-miR-3202, hsa-miR-4260, hsa-miR-4267, hsa-miR-3661, hsa-miR-3180, hsa-miR-4476, hsa-miR-4478, etc.
PSAT1	hsa-miR-124-5p, hsa-miR-18b-5p, hsa-miR-125b-5p, hsa-miR-2115-5p, hsa-miR-6838-5p, hsa-miR-16-5p, hsa-miR-125b-5p, hsa-miR-18b-5p, hsa-miR-504-5p, hsa-miR-2115-5p, hsa-miR-6838-5p

### Immune infiltration landscape analysis

**Figure 8:**
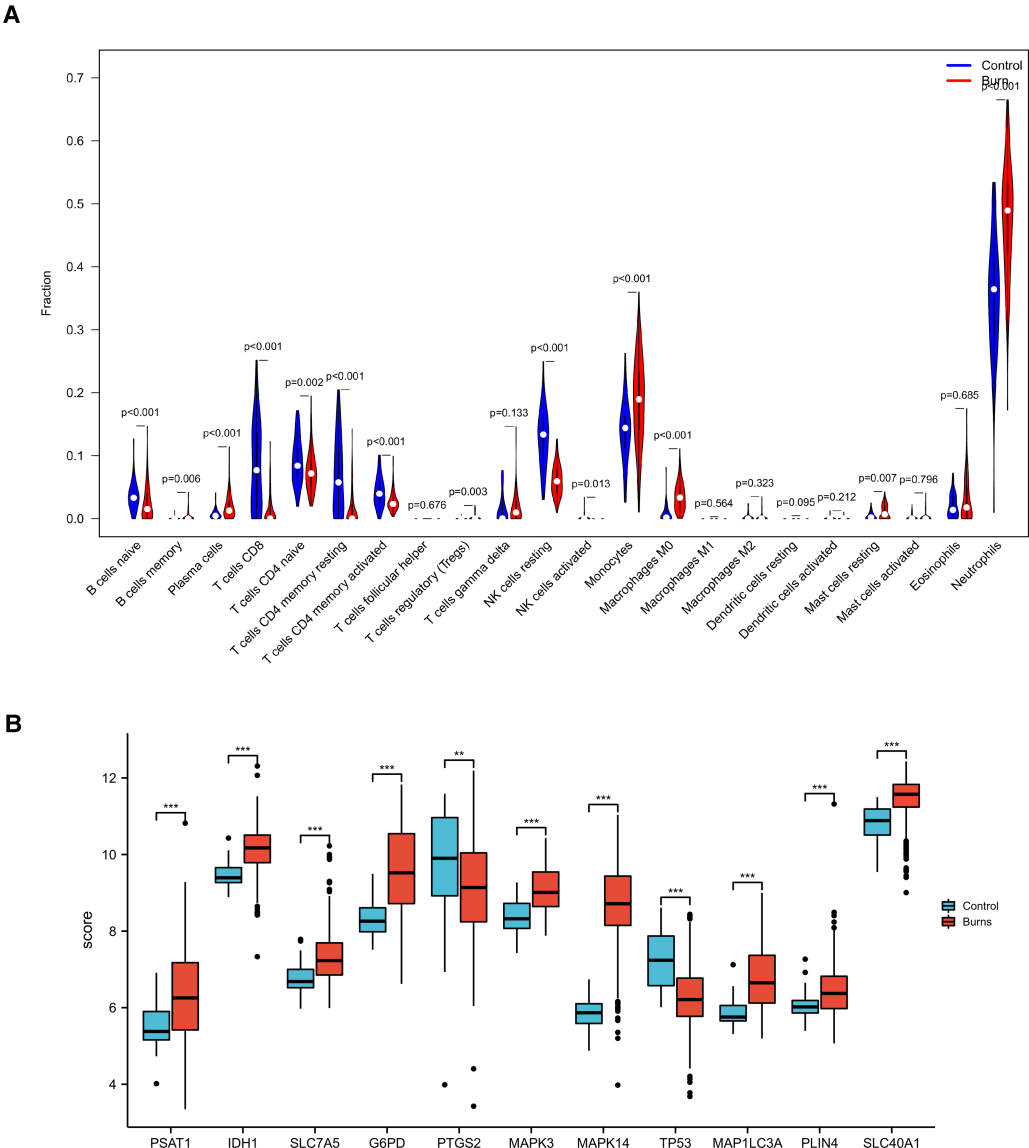
(**A**) CIBERSORT immuno-infiltration analysis of burn patients and controls (**B**) Hub gene expression in the validation series.

The control (healthy) group showed significantly higher levels of T, NK, and B primitive cells compared with the burn group. The burned individuals showed significantly higher levels of monocytes, macrophages, mast cells, neutrophils, plasma cells, and T cells gamma delta, which was consistent with our cognition (Figure 8A) (22).

## Discussion

Ferroptosis is a form of programmed necrosis primarily triggered by iron-dependent extra-mitochondrial lipid peroxidation and ROS accumulation (23). In this study, we identified hub genes of ferroptosis and explored their molecular mechanisms in burns. We screened 64 FDEGs by the intersection of the databases of DEGs in GSE19743 and FerrDb, and we selected 10 hub genes of interest. Enrichment analysis through GO-BP shows significant involvement of these genes in oxidative stress, pyridine-containing compound metabolic process, and reactive oxygen species metabolic process. Although, to our knowledge, there is no exact experimental evidence of ferroptosis in burns, ROS accumulation and lipid peroxidation can be observed in the serum of burn patients (24, 25). Antioxidants were shown to significantly reduce injury and edema after burns (26–28). Furthermore, the use of metal chelation can decrease oxidation and inflammation, and alleviate burn injury (29, 30). It has been reported that the endothelial permeability increases in a time-dependent manner after the burn, parallel to the rapid and continuous activation of p38 MAP kinase (31). p38 MAPK is specifically involved in actin and myosin rearrangements (stress-fiber formation) in endothelial cells causing cell contraction and enhancement of vascular permeability (32). Inhibition of p38 MAPK was shown to significantly ameliorate burn-induced vascular dysfunction (33).

MicroRNAs are widely used as biomarkers for disease prevention, diagnosis, and prognosis (34). In this study, through miRNA enrichment analysis, we found significantly enriched molecular functions, namely, signal transduction, cell communication, and regulation of nucleobase, nucleoside, nucleotide, and nucleic acid metabolism. To this date, several miRNAs have been described as modulators of burn injury pathogenesis and healing. Upon a burn injury, MiR-24-3p, regulated by MAPK14, promotes inflammatory cytokine expression through PPAR-β modulation, to inhibit migration and proliferation of heat-damaged human skin fibroblasts (35). MiR-16-5p activates the p38 MAPK pathway by targeting bridging granule core protein 3, thus promoting *in vitro*-induced keratin-forming cell migration and burn wound re-epithelialization and healing. This offers new perspectives for miRNA-mediated therapeutic strategies in burns (36). Furthermore, FDEGs were found to be enriched in the VEGF signaling pathway through KEGG pathway analysis. VEGF rapid increase after burns is an important mediator of angiogenesis and tissue repair, regulating vascular permeability and adhesion between monocytes and endothelial cells (37, 38). In the early stages of scarring, fibroblasts produce large amounts of VEGF, transforming growth factor-beta, collagen I, and collagen III causing tissue proliferation (39). Moreover, the occurrence of serious complications during the post-traumatic period (such as sepsis, respiratory distress syndrome, and multiple organ failure) is associated with a dampened VEGF increase after injury (40).

PTGS2, also known as cyclooxygenase-2 (COX-2), is an inflammatory mediator that takes part in membrane phospholipid metabolism and prostaglandin synthesis (41). The use of COX-2 inhibitors prevents the inflammatory response, promotes wound healing, and reduces keloid formation (42). Mechanical injury can regulate COX-2-dependent prostaglandin E2 production in a time-dependent manner. This triggers ROS production in keratinocytes and consequent inflammatory response in the wound (43). Furthermore, COX-2 regulates body fat and the synthesis of uncoupling protein 1 (UCP1), which activates brown adipose tissue and hypermetabolism. Therefore, COX-2 might be responsible for the hypermetabolic state after burns (44). This hypermetabolic response persists after the burn injury and promotes severe muscle and protein catabolism, insulin resistance, and cardiac insufficiency. This state is characterized by high levels of catecholamines, glucagon, and cortisol, which increase mortality in burn patients (45, 46). Considering other prognosis factors, we identified genes that can be used as biomarkers for this purpose and eventually determine therapeutic approaches.

In this study, we identified two prognostic-related genes, SLC40A1 and GPT2, correlated with overall survival. Ferroportin, also known as SLC40A1, is a member of the solute carrier family and transports iron from the duodenum, spleen, and macrophages to the liver cells for storage. Thus, this protein is critical for iron homeostasis (47). Extensive burns cause anemia and critical illness, and the only therapeutic alternative is blood transfusion since erythropoietin and iron supplements fail to promote effective erythropoiesis. Burn injury induces an macrophage colony-stimulating factor (M-CSF)-dependent iron recycling program in the liver and spleen, and iron transporter expression recovered over time. This may be an iron homeostasis response to the increased destruction of red blood cells (48). Meanwhile, GSEA showed that SLC40A2 enrichment was immune-related, including pathways of antigen processing and presentation (the most significant downward adjustment in GSVA), graft vs. host disease, the intestinal immune network for IgA production, NK cell-mediated cytotoxicity, etc. GPT2, also known as alanine transaminase 2, produces α-ketoglutarate and alanine by catalyzing the reversible addition of amino groups from glutamate to pyruvate (49). GSEA revealed GPT2 enrichment in the pentose and glucuronate interconversions pathway. Martino et al. found that GPT2 knockout in the diabetic liver reduced amino acid gluconeogenesis and decreased hepatic pyruvate utilization (50). GPT2 maintains tricarboxylic acid cycle anaplerosis after glutaminase (GLS) inhibition, contributing to cell survival and growth. GPT2 inhibition results in reduced cell proliferation and increased cell death (51). This may explain why high GPT2 expression correlates with a higher survival outcome in our results. The identified prognostic genes can provide indications and clinical directions for burn treatment.

Previous studies have demonstrated a decrease in NK cells following burns (52). Collectively, data seem to suggest that severe burns cause a sustained innate inflammatory response; with the release of immature neutrophils and a shift in T-cell composition toward a proinflammatory phenotype, resulting in persistent systemic inflammation and increased risk of secondary complications (53). T cells gamma delta regulate bone marrow cell infiltration at the wound site and act to quell inflammation, thus facilitating the transition from wound healing to a proliferative phase (54). This suggests the importance of mast cell signaling in the effective recruitment and migration of additional immune cells to the site of injury. Notably, mast cells are not only present in the early stages of the immune system response to burns. Meanwhile, the role of B cells may be downregulated after thermal injury (22). In our study, these phenomena were well confirmed.

Although we believe our results may have a significant impact on the field, our study presented some limitations. First, the sample size used for this study was small, which might lead to relevant bias. In addition, burns’ prognosis can be affected by multiple factors such as the burn area, shock, and inhalation injury that were not discussed here and will be further deepened in subsequent studies.

Overall, our study is the first to report an FDEG expression signature in burns, which demonstrates that ferroptosis is an important cell death type in these injuries. As a recently described cell death type, it may broaden the therapeutic direction of burns. Enrichment analysis of FDEGs and its associated miRNA highlight new underlying mechanisms in burns. Finally, the identification of prognosis will be able to improve treatment decisions of burned patients and prevent the emergence of poor prognostic states.

## Conclusion

In this study, we identified 10 hub genes (IDH1, GLS2, PTGS2, MAPK3, MAPK14, TP53, MAP1LC3A, G6PD, PSAT1, and SLC7A5) and analyzed them through enrichment analysis, PPI network, molecular correlation, gene–miRNA network, and miRNA enrichment. In addition, we further identified potential prognosis-related genes (SLC40A1 and GPT2), that might be closely related to a poor prognosis after burn injury. This study described the link between ferroptosis and burn injury, providing a basis for the future development of related drugs and new therapeutic options. Moreover, we observed that genetic changes after burn can reflect immune cells’ performance, highlighting new targets for future research in burn patients.

## Data Availability

The datasets presented in this study can be found in online repositories. The names of the repository/repositories and accession number(s) can be found here: https://www.ncbi.nlm.nih.gov/geo/query/acc.cgi?acc=GSE19743.
